# Analysis of miRNA Expression Profiling of *RIP2* Knockdown in Chicken HD11 Cells When Infected with Avian Pathogenic *E. coli* (APEC)

**DOI:** 10.3390/ijms23137319

**Published:** 2022-06-30

**Authors:** Hongyan Sun, Yuxuan Cao, Yexin Yang, Huan Li, Lujiang Qu

**Affiliations:** 1 College of Animal Science and Technology, Yangzhou University, Yangzhou 225009, China; yxc@126.com (Y.C.); YYang@163.com (Y.Y.); 2 Joint International Research Laboratory of Agriculture & Agri-Product Safety, Ministry of Education, Yangzhou University, Yangzhou 225009, China; 3 School of Biological and Chemical Engineering, Yangzhou Polytechnic College, Yangzhou 225009, China; 4 College of Animal Science and Technology, China Agricultural University, Beijing 100091, China; qulujj@163.com

**Keywords:** avian pathogenic *E. coli*, miRNAs, *RIP2*, gga-miR-455-5p, inflammatory cytokine, *IRF2*

## Abstract

Colibacillosis is an acute and chronic avian disease caused by avian pathogenic *E. coli* (APEC). Previous studies have demonstrated that *RIP2* plays a significant role in APEC infection. Moreover, increasing evidence indicates that microRNAs (miRNAs) are involved in host–pathogen interactions and the immune response. However, the role of miRNAs in the host against APEC infection remains unclear. Herein, we attempted to reveal new miRNAs potentially involved in the regulation of the immune and inflammatory response against APEC infection, with a particular focus on those possibly correlated with *RIP2* expression, via miRNA-seq, RT-qPCR, Western blotting, dual-luciferase reporter assay, and CCK-8. The results showed that a total of 93 and 148 differentially expressed (DE) miRNAs were identified in the knockdown of *RIP2* cells following APEC infection (shRIP2+APEC) vs. knockdown of *RIP2* cells (shRIP2) and shRIP2 vs. wild-type cells (WT), respectively. Among those identified DE miRNAs, the biological function of gga-miR-455-5p was investigated. It was found that gga-miR-455-5p regulated by *RIP2* was involved in the immune and inflammatory response against APEC infection via targeting of *IRF2* to modulate the expression of type I interferons. Additionally, *RIP2* could directly regulate the production of the type I interferons. Altogether, these findings highlighted the crucial role of miRNAs, especially gga-miR-455-5p, in host defense against APEC infection.

## 1. Introduction

Avian pathogenic *E. coli* (APEC) can cause acute and chronic diseases in chickens, ducks, geese, turkeys, and other birds, including pericarditis, airsacculitis, perihepatitis, septicemia, meningitis, etc., and is collectively known as avian colibacillosis. APEC is not only detrimental to the health of birds [1], but also affects egg production [2], delays the time to market of broilers [2], and increases the feeding cost [2,3], resulting in great losses to the poultry industry and posing a great threat to food safety. Previous studies have shown that avian and human *E. coli* had high homology and similarity in genome structure, genetic evolution, and ecological distribution [4]. Different extraintestinal pathogenic *E. coli* have common virulence-related genes, similar disease patterns, and phylogenetic backgrounds [5]. Evidence is increasingly showing that APEC poses a greater potential threat to human health. Therefore, it is of greater practical significance to strengthen the research on APEC and host immune response.

Many immune-related signaling pathways were activated in the primary and secondary immune tissues upon APEC infection when using whole transcriptome analysis [6,7,8,9,10]. In particular, the NOD-like receptor signaling pathway and its core gene *RIP2* (receptor interacting serine/threonine kinase 2) were identified to be a common transcriptomic immune response to APEC stimulation [6,7,8]. RIP2 is the master regulator of inflammatory response, and its expression and activation are tightly regulated at several levels [9,10,11]. In addition to bacterial infection, *RIP2* also has the ability to mediate the response to influenza A virus (IAV) infection and DNA damage [12,13]. Moreover, a deficiency in *RIP2* will result in an increase in mitochondrial damage upon IAV infection, further reducing ULK1 phosphorylation and mitophagy [14]. While researchers have performed extensive studies on the function of *RIP2*, the specific regulation mechanism of *RIP2* is not fully understood at the transcriptomic level.

MiRNA is a type of noncoding single-strand small RNA that regulates the target mRNA at the post-transcriptional level [15,16]. Numerous miRNAs have been reported to participate in the regulation of pathological and physiological processes as important regulators of bacterial infection. For example, miR-146, miR-155, miR-125, let-7, and miR-21 are certainly involved in immune responses upon bacterial infection, such as with *Mycobacterium tuberculosis* [17,18], *Helicobacter pylori* [19,20], and *Listeria monocytogenes* [21]. In particular, miR-17-5p has a function to regulate autophagy upon Mycobacteria infection in murine macrophages [22]. Moreover, let-7i-3p miRNA can strongly inhibit *Salmonella* replication via modulation of endolysosomal trafficking and the vacuolar environment by targeting the host RGS2 protein [23]. Overexpression of gga-miR-429 can significantly inhibit the expression of TMEFF2 and SHISA2 to further regulate the platelet-derived growth factor (PDGF) and Wnt signaling pathways following APEC infection in chicken HD11 macrophages [24]. In addition, Zhao et al. found that miR-200c-3p can suppress lipopolysaccharide (LPS)-induced inflammatory responses by targeting *RIP2* [25]. However, in general, little is known about the regulatory mechanisms used by miRNAs in APEC infection, and very few studies have focused on those correlated with *RIP2*.

The specific aim of this study was to evaluate new miRNAs potentially involved in APEC infection, with a particular focus on those that were correlated with *RIP2* expression, by profiling miRNAs in normal HD11 cells, as well as APEC-stimulated and *RIP2*-knockdown HD11 macrophages, using RNAseq and luciferase assays. 

## 2. Results

### 2.1. Characteristics and High-Throughput Sequencing of Small RNA

We constructed 12 small RNA libraries representing the complete HD11 cells (WT, *n* = 3), *RIP2* knockdown of HD11 cells (shRIP2, *n* = 3), APEC-infected HD11 cells (APEC, *n* = 3), and APEC-infected *RIP2*-knockdown HD11 cells (shRIP2+APEC, *n* = 3) groups, which were sequenced to identify miRNAs involved the immune response of chicken HD11 cells to APEC infection with or without *RIP2* knockdown. The sequencing generated raw reads as shown in Table S1. The total raw sequencing reads reached 250,142,786 with an average of 20,845,232 reads per sample. After removing the low-quality raw reads, sequences containing N, and length anomalies, there were 125,004,269 clean reads with an average of 10,417,022 reads per sample (Table S1). Among the 12 samples, the sequence length distribution exhibited a wide variation, ranging from 18 to 30 nucleotides (Figure S1A). The most abundant classes of small RNAs for the four treatments were 20-24 nucleotides in length, predominantly 22 nucleotides, which indicated that the sequencing results were consistent with the miRNA length distribution. Moreover, compared to the wild-type HD11 cells (average rate = 45.73%), the APEC infection group had the largest proportion of 22 nucleotides (average rate = 51.51%), followed by the *RIP2*-knockdown (average rate = 48.28%) and APEC-infected *RIP2*-knockdown-cell groups (average rate = 47.17%).

**Figure 1:**
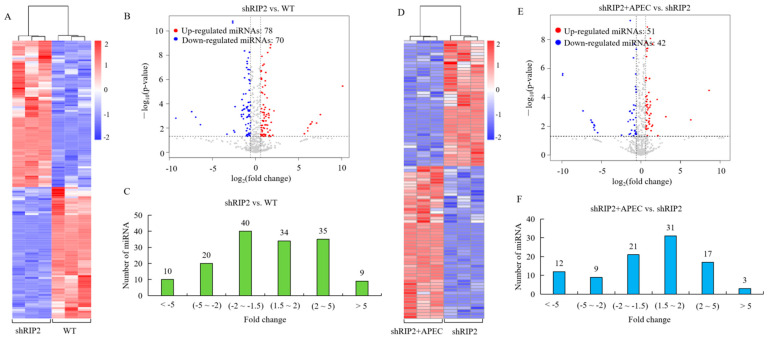
The miRNA-seq profiling in the comparisons of the *RIP2*-knockdown HD11 cells group (shRIP2) vs. the wild-type cells group (WT) and *RIP2*-knockdown HD11 cells combined with the APEC infection group (shRIP2+APEC) vs. shRIP2. (**A**,**D**) Heatmap analysis for the miRNA-seq data from the comparison of shRIP2 vs. WT (**A**) and shRIP2+APEC vs. shRIP2 (**D**). Red color indicates upregulation, while blue means downregulation. (**B**,**E**) The expression levels of differentially expressed miRNAs in the comparison of shRIP2 vs. WT (**B**) and shRIP2+APEC vs. shRIP2 (**E**). Red spots represent DE miRNAs for upregulation, blue spots represent downregulation, and grey spots represent unchanged miRNAs. (**C**,**F**) The distribution of the differentially expressed miRNAs in the comparison of shRIP2 vs. WT (**C**) and shRIP2+APEC vs. shRIP2 (**F**).

The clean reads were then mapped to the *Gallus gallus 6.0* reference genome and Rfam to annotate the categories of the noncoding RNAs. As shown in Table S2, the average mapping rate of the 12 small RNA sequencing libraries was 96.68% (83,319,613/86,182,552), while the average unique mapping rate was 87.74% (2,397,108/2,732,025), indicating that most of the sequencing data were chicken miRNA. The percentage for each rRNA, tRNA, snRNA, snoRNA, and novel miRNA among the 12 samples was less than 3.5% in the mapping results, whereas known miRNA (>60%) was the predominant category. It was worth noting that APEC infection had the largest proportion of known miRNAs (67.55%), followed by *RIP2*-knockdown HD11 cells (66.07%), APEC-infected *RIP2*-knockdown HD11 cells (62.88%), and wild-type HD11 cells (60.6%). The distribution of reads on functional elements of the reference genome for the four groups were mainly concentrated in intron (average rate = 27.45%) and intergenic (average rate = 29.03%), followed by promoter (average rate = 21.33%) and 5’ untranslated region (5’ UTR, average rate = 15.1%) (Figure 1B), of which the wild-type HD11 cells had the largest number of those miRNAs in each region, followed by the *RIP2*-knockdown HD11 cells, APEC-infected *RIP2*-knockdown HD11 cells, and APEC infection group. The density of reads of the four groups were distributed on chromosomes 1-15, 17-19, 27, 33, and Z (Figure S1C). It was interesting that no sequences were mapped to chromosome W.

### 2.2. Known and Novel miRNA Analysis

After comparing the mapped reads with the specific range of the sequence in miRBase, we found that knockdown of *RIP2* HD11 cells had the largest number of known hairpin and mature miRNAs, followed by the wild-type HD11 cells, APEC infection group, and *RIP2*-knockdown HD11 cells combined with the APEC infection group. Due to the development of miRNA from precursor to mature being conducted with the assistance of Dicer, we also identified the strong bias of the first base of the miRNA mature sequence for the specificity of the Dicer restriction site by calculating the occurrence frequency. In general, the first base of A was the strongest preference in 27- and 28-nucleotide-long miRNA (Figure S2A). The first base of U was the predominant preference for 20-23 and 26 nucleotides. Moreover, the miRNAs had a major preference for U at position 1. The base preferences were roughly equivalent at other positions (Figure S2B).

The unannotated sequences from the chicken small RNA library were then mapped to the chicken genome database to predict the potential novel miRNAs. A total of 128,898 miRNAs were detected to align against chicken genome, of which an average of 244 novel miRNAs were identified to unevenly express, with a length ranging from 18 to 25 nucleotides. The first base of U was the predominant bias for the size of 19 to 25 nucleotides (Figure S2C). Generally, the base bias at each position, except position 1 (predominant with base U), was performed approximately equally in each of those detected miRNAs (Figure S2D).

### 2.3. Analysis of Differentially Expressed (DE) miRNA

To identify the DE miRNAs in wild-type HD11 cells and *RIP2*-knockdown HD11 cells with or without APEC infection, the normalized expressions of the miRNAs in each group were compared. A cluster analysis of the DE miRNAs (148 in shRIP2 vs. WT and 93 in shRIP2+APEC vs. APEC) was performed (Figure 1A,D), indicating that the upregulated and downregulated miRNAs may have had similar functions or participated in the same biological process. Volcano maps of DE miRNAs (Figure 1B,E) and graphs of the fold-change distribution of DE miRNAs (Figure 1C,F) were drawn. In general, there were 93 (42 downregulated and 51 upregulated) and 148 (70 downregulated and 78 upregulated) DE miRNAs in the comparison of shRIP2+APEC vs. shRIP2 and shRIP2 vs. WT, respectively. Among them, 54 (17 downregulated and 37 upregulated) and 39 (23 downregulated and 16 upregulated) novel DE miRNAs were identified in the aforementioned two comparisons, respectively. Compared to the *RIP2*-knockdown group, 42 DE miRNAs were downregulated in the knockdown of *RIP2* combined with APEC infection (19 known and 23 novel), whereas 51 (35 known and 16 novel) miRNAs were upregulated (Figure 1E,F). The *RIP2*-knockdown group had 70 downregulated DE miRNAs (53 known and 17 novel) and 78 upregulated (41 known and 37 novel) in comparison to the wild-type HD11 cells (Figure 1B,C). These findings indicated that the identified known and novel DE miRNAs might have specific but vital function in different conditions.

### 2.4. Analysis of Potential Target Genes of Differentially Expressed (DE) miRNAs

The identified known and novel DE miRNAs were subsequently used to explore the corresponding potential target genes. A total of 15,239 putative miRNA targets were detected for 739 known miRNAs, while 15,338 miRNA target genes were found for 981 novel miRNAs. The numbers of potential target genes for each miRNA ranged from 1 to 3045. In general, except for gga-miR-1812-3p and gga-miR-190a-3p, all the miRNAs had more than one target, of which 45.58% (784/1720) of the miRNAs had 100~500 targets, followed by 20% (344/1720) miRNAs with 500~1000 targets. These results indicated that the identified miRNAs were functionally divergent.

**Figure 2:**
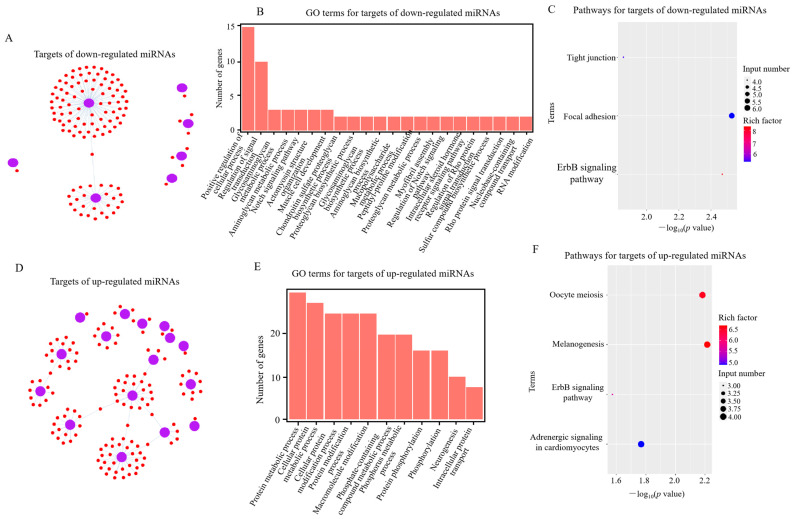
Analysis of potential target genes of differentially expressed (DE) miRNAs in *RIP2*-knockdown combined with APEC infection HD11 cells (shRIP2+APEC) vs. *RIP2*-knockdown HD11 cells (shRIP2). (**A**,**D**) Targets of down- (**A**) and upregulated (**D**) DE miRNAs in shRIP2+APEC vs. shRIP2. (**B**,**E**) GO-term analysis of target genes of down- (**B**) and upregulated (**E**) DE miRNAs in shRIP2+APEC vs. shRIP2. (**C**,**F**) KEGG-pathway analysis of target genes of down- (**C**) and upregulated (**F**) DE miRNAs in shRIP2+APEC vs. shRIP2.

**Figure 3:**
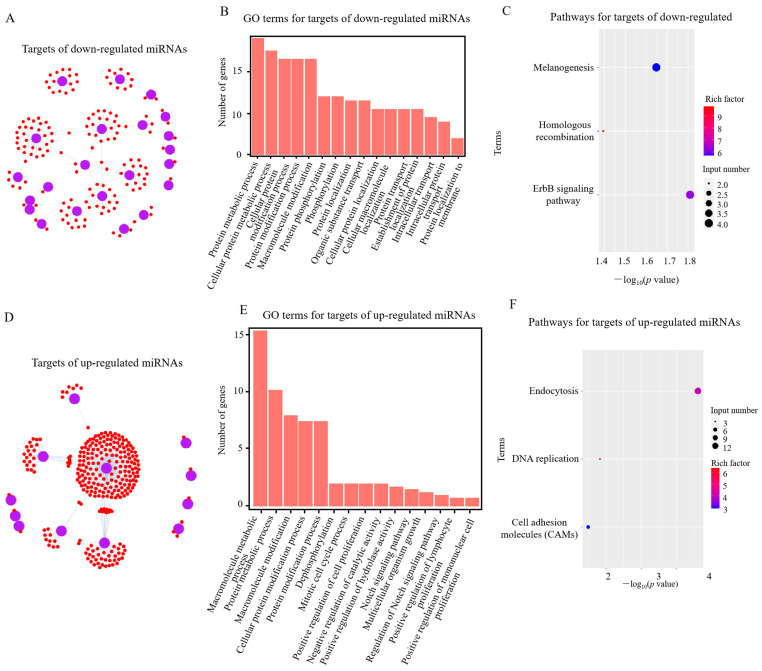
Analysis of potential target genes of differentially expressed (DE) miRNAs in *RIP2*-knockdown HD11 cells (shRIP2) vs. wild-type HD11 cells (WT). (**A**,**D**) Targets of down- (**A**) and upregulated (**D**) DE miRNAs in shRIP2 vs. WT. (**B**,**E**) GO-term analysis of target genes of down- (**B**) and upregulated (**E**) DE miRNAs in shRIP2 vs. WT. (**C**,**F**) KEGG-pathway analysis of target genes of down- (**C**) and upregulated (**F**) DE miRNAs in shRIP2 vs. WT.

Compared with the *RIP2*-knockdown group, there were 116 target genes corresponding to 7 downregulated miRNAs and 136 targets for 16 upregulated miRNAs in the *RIP2* knockdown combined with APEC infection group (Figure 2A,D). In the comparison of *RIP2*-knockdown HD11 cells vs. wild-type HD11 cells, 165 target genes for 21 downregulated miRNAs and 302 targets for 12 upregulated miRNAs were identified (Figure 3A,D). Moreover, in the comparison of shRIP2+APEC vs. shRIP2, the putative target genes of the downregulated miRNAs were found to be involved in the positive regulation of cellular processes, the regulation of signal transduction, the Notch signaling pathway, and regulation of the Notch signaling pathway gene ontology (GO) terms, whereas the targets of the upregulated miRNAs were responsible for protein metabolic processes, macromolecule modification, and phosphorylation (Figure 2B,E). In the comparison of shRIP2 vs. WT, the predicted target genes of down- and upregulated miRNAs in the category of biological processes (Figure 3B,E) were similar to those GO terms in shRIP2+APEC vs. shRIP2.

Following the GO analysis, the Kyoto Encyclopedia of Genes and Genomes (KEGG) was used to detect the pathway enrichment of the predicted miRNA target genes. In the comparison of shRIP2+APEC vs. shRIP2, the downregulated DE miRNA targets were involved in tight junctions, focal adhesion, and the ErbB signaling pathway, while those upregulated were involved in adrenergic signaling in cardiomyocytes, the ErbB signaling pathway, melanogenesis, and oocyte meiosis (Figure 2C,F). In addition, the ErbB signaling pathway was also enriched in the downregulated DE miRNAs targets between shRIP2 and WT (Figure 3C), indicating the important role of this pathway. However, the upregulated DE miRNAs targets between shRIP2 and WT were abundant in endocytosis, DNA replication, and cell-adhesion molecules (Figure 3F).

### 2.5. Verification of miRNAs by RT-qPCR

**Figure 4:**
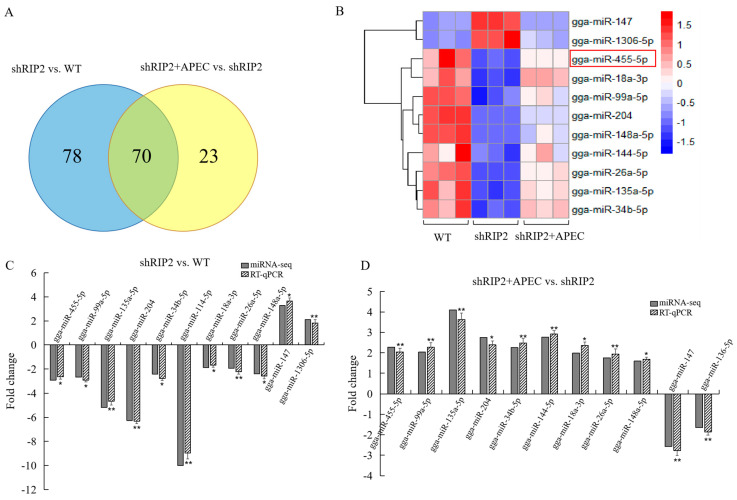
Verification of miRNA-seq data by RT-qPCR for the comparison of *RIP2*-knockdown HD11 cells (shRIP2) vs. wild-type HD11 cells (WT) and *RIP2*-knockdown cells combined with APEC infection (shRIP2+APEC) vs. shRIP2. (**A**) Venn analysis of differentially expressed miRNAs in shRIP2 vs. WT and shRIP2+APEC vs. shRIP2. (**B**) The heatmap of the selected differentially expressed miRNAs in WT, shRIP2, and shRIP2+APEC. (**C**,**D**) The relative expression of the selected differentially expressed miRNAs according to RT-qPCR in shRIP2 vs. WT (**C**) and shRIP2+APEC vs. shRIP2 (**D**). Data are shown as mean ± SD; *n* = 3 independent experiments; * *p* < 0.05, ** *p* < 0.01.

A total of 70 DE miRNAs were identified in both shRIP2+APEC vs. shRIP2 and shRIP2 vs. WT (Figure 4A), of which 11 DE miRNAs were arbitrarily chosen for qRT-PCR to validate the reliability of the miRNA-seq data; these are displayed in the heatmap in Figure 4B. These selected DE miRNAs included gga-miR-455-5p, gga-miR-99a-5p, gga-miR-135a-5p, gga-miR-204, gga-miR-34b-5p, gga-miR-147, gga-miR-144-5p, gga-miR-1306-5p, gga-miR-18a-3p, gga-miR-148a-5p, and gga-miR-26a-5p (Figure 4B), of which gga-miR-147 and gga-miR-1306-5p had a significant high expression in the shRIP2 group compared with the WT and shRIP2+APEC group, while the other nine DE miRNAs had a significantly low expression in the shRIP2 group compared with the WT and shRIP2+APEC group. The results showed that the miRNAs found in the RT-qPCR were in high agreement with the miRNA-seq data on the basis of identifying the up- and downregulated miRNAs (Figure 4C,D).

### 2.6. APEC Infection Affected the Expression of gga-miR-455-5p, RIP2, IRF2, IFNɑ, and IFNβ

**Figure 5:**
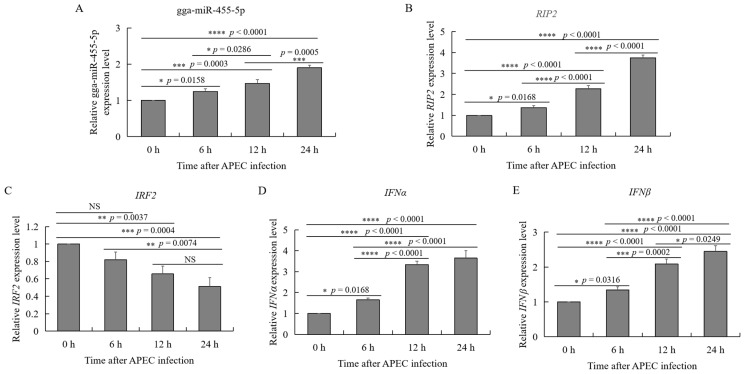
Effects of APEC infection on gga-miR-455-5p, *RIP2*, *IRF2*, *IFNα*, and *IFNβ* expression in chicken HD11 cells. (**A**–**E**) The relative expression of miRNAs, including gga-miR-455-5p (**A**), *RIP2* (**B**), *IRF2* (**C**), *IFNα* (**D**), and *IFNβ* (**E**) in HD11 cells after infection with APEC at a cfu of 10^8^ for 6, 12, and 24 h as determined by qRT-PCR. Data are shown as mean ± SD; *n* = 3 independent experiments; * *p* < 0.05, ** *p* < 0.01, *** *p* < 0.001, **** *p* < 0.0001.

To investigate the relationship between gga-miR-455-5p and *RIP2* in vitro during APEC infection, RT-qPCR was performed to determine the expression of gga-miR-455-5p and *RIP2* in HD11 cells after 6, 12, and 24 h of APEC challenge. As shown in Figure 5A and B, APEC infection significantly increased the expression of gga-miR-455-5p and *RIP2* in a time-dependent manner. These results suggested that both gga-miR-455-5p and *RIP2* expression were upregulated during APEC infection. As we predicted, *IRF2* was a candidate target of gga-miR-455-5p, and *IRF2* could regulate the production of type I interferons (IFNα/β). The expression levels of *IRF2*, *IFN*α, and *IFNβ* were also identified after APEC infection. The results showed that APEC infection significantly inhibited the expression of *IRF2*, whereas it increased the expression of *IFN*α and *IFNβ* in a time-dependent manner (Figure 5C–E).

### 2.7. Biological Function of gga-miR-455-5p in Chicken HD11 Cells after APEC Infection

**Figure 6:**
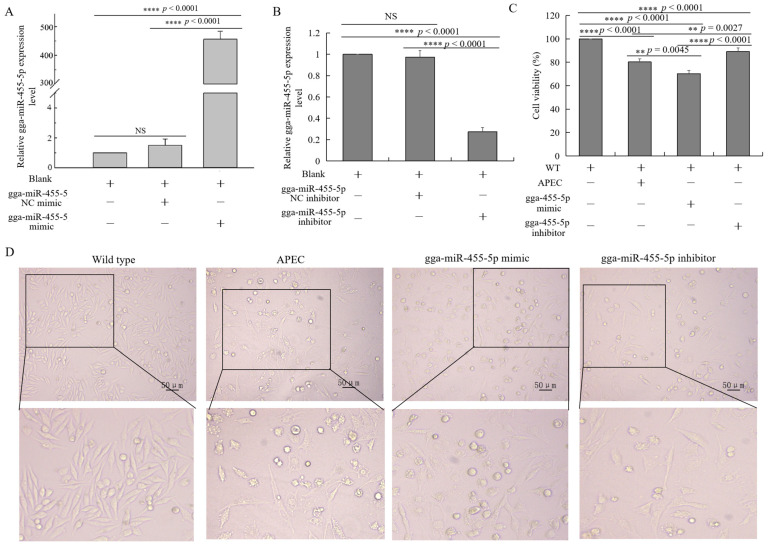
Effect of gga-miR-455-5p on cell survival. (**A**,**B**) The relative expression of gga-miR-455-5p in HD11 cells transfected with gga-miR-455-5p mimic (**A**) or inhibitor (**B**) for 48 h (data are shown as mean ± SD; *n* = 3 independent experiments; **** *p* < 0.0001; NS, not significant). (**C**) The cell viability of chicken HD11 macrophages in the wild-type, APEC infection, gga-miR-455-5p mimic, and gga-miR-455-5p inhibitor groups (data are shown as mean ± SD; *n* = 3 independent experiments; ** *p* < 0.01, **** *p* < 0.0001). (**D**) The morphology of chicken HD11 macrophages in the wild-type, APEC infection, gga-miR-455-5p mimic, and gga-miR-455-5p inhibitor groups.

To further examine the effects of gga-miR-455-5p on APEC infection, a gga-miR-455-5p mimic and inhibitor were constructed to overexpress or knock down the endogenous gga-miR-455-5p expression in HD11 cells. At 48 h after transfection, the expression of gga-miR-455-5p was markedly increased in HD11 cells transfected with the gga-miR-455-5p mimic in comparison to the blank and mimic negative control group (Figure 6A). Compared with the blank and inhibitor negative control, the expression of gga-miR-455-5p was significantly suppressed in HD11 cells at 48 h after transfection with gga-miR-455-5p inhibitors (Figure 6B).

**Figure 7:**
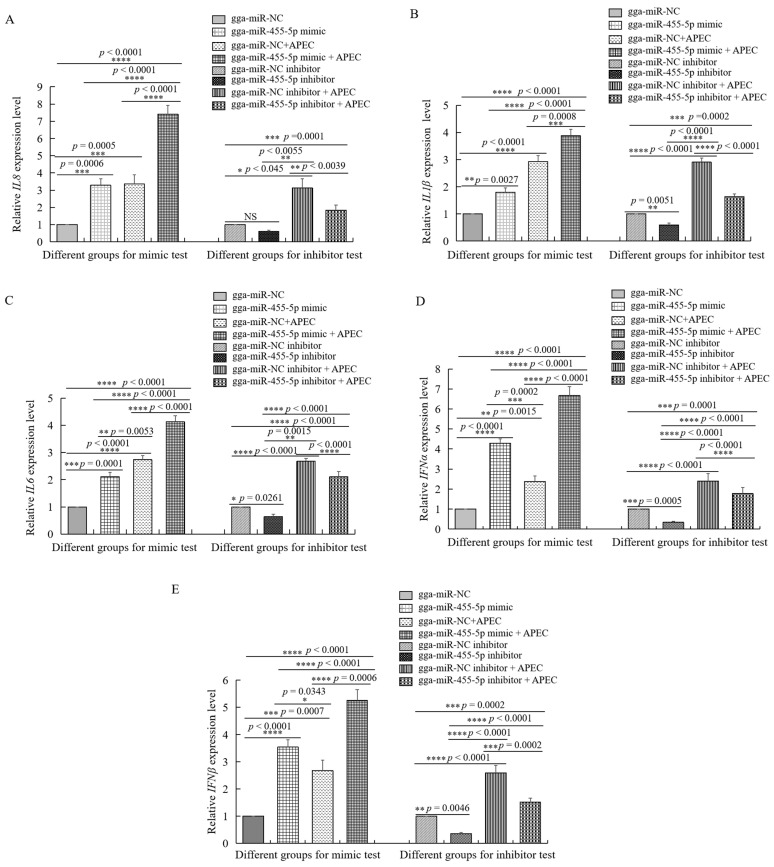
The effects of gga-miR-455-5p on different cytokines with or without APEC infection. (**A**–**E**) The expression levels of five proinflammatory mediators, including *IL8* (**A**), *IL1β* (**B**), *IL6* (**C**), *IFNα* (**D**), and *IFNβ* (**E**) were analyzed using RT-qPCR after transfection with the gga-miR-455-5p mimic or negative control and gga-miR-455-5p inhibitor or control inhibitor with or without APEC infection. Data are shown as mean ± SD; *n* = 3 independent experiments; * *p* < 0.05, ** *p* < 0.01, *** *p* < 0.001, **** *p* < 0.0001.

At 48 h after transfection with the gga-miR-455-5p mimic or inhibitor, HD11 cells were infected with APEC for 24 h. As shown in Figure 6C,D, the cell survival was significantly increased in the gga-miR-455-5p inhibitor group, while it decreased in the gga-miR-455-5p mimic group. Furthermore, we examined the expression of *IL8*, *IL1β*, *IL6*, *IFNα*, and *IFNβ* with or without APEC infection after transfection with the gga-miR-455-5p mimic or inhibitor at 48 h. The results showed that APEC challenge promoted the significant upregulation of cytokines and induced the release of inflammatory mediators in HD11 cells (Figure 7AE). The inhibition of endogenous gga-miR-455-5p decreased the expression of *IL8*, *IL1β*, *IL6*, *IFNα*, and *IFNβ* with or without APEC infection (Figure 7A–E). However, their expression levels were increased in the gga-miR-455-5p mimic group with or without APEC infection (Figure 7A–E). These results clearly established the role of gga-miR-455-5p as an antibacterial factor in the cellular response against APEC infection.

### 2.8. IRF2 Was the Target of gga-miR-455-5p

**Figure 8:**
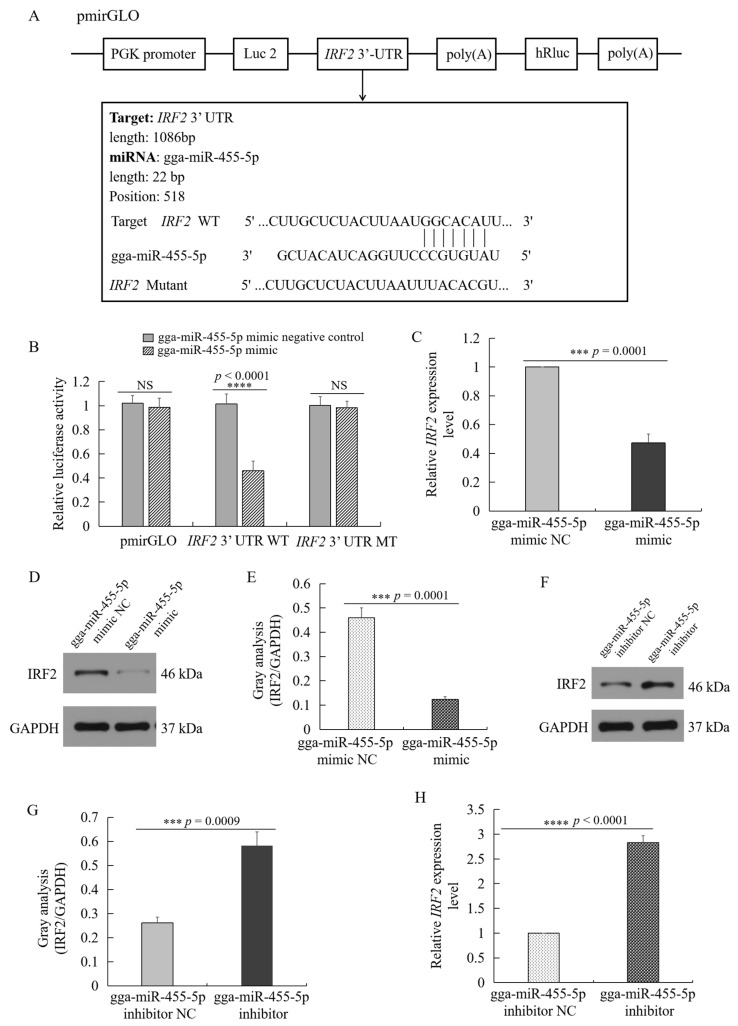
*IRF2* is a target of gga-miR-455-5p. (**A**) The sequence alignment of gga-miR-455-5p and one target site in the 3’UTR of *IRF2* are shown. (**B**) HD11 cells were cotransfected with pmirGLO or *IRF2* 3’ UTR luciferase reporter plasmid (wild-type or mutation), along with control mimics or gga-miR-455-5p mimics as indicated. Firefly luciferase activity was measured and normalized using Renilla luciferase activity to finally calculate the relative luciferase activity 48 h after transfection. (**C**,**H**) The mRNA expression level of *IRF2* in HD11 cells transfected with gga-miR-455-5p mimic (**C**) or inhibitor (**H**) (data are shown as mean ± SD; *n* = 3 independent experiments; *** *p* < 0.001, **** *p* < 0.0001). (**D**,**F**) The protein expression of *IRF2* in HD11 cells transfected with gga-miR-455-5p mimic (**D**) or inhibitor (**F**). (**E**,**G**) Image J software was used for IRF2 gray-level analysis of Western blot results in HD11 cells transfected with gga-miR-455-5p mimic (**E**) or inhibitor (**G**) (data are shown as mean ± SD; *n* = 3 independent experiments; *** *p* < 0.001).

According to the results of miRanda, a target scan, and miRDB, *IRF2* was predicted to be the potential target of gga-miR-455-5p with the highest score. Then, the molecular mechanism underlying gga-miR-455-5p was investigated by using the luciferase reporter gene system. The 3’ UTRs of *IRF2* were cloned into the luciferase reporter plasmid to identify the function of gga-miR-455-5p in chicken HD11 cells (Figure 8A). The potential binding site in the 3’ UTR of *IRF2* was found for gga-miR-455-5p (Figure 8A). Additionally, the dual-luciferase reporter assays on HD11 cells 48 h after cotransfection were performed to validate the binding of gga-miR-455-5p with the candidate target *IRF2*. As shown in Figure 8B, the gga-miR-455-5p mimics significantly inhibited the luciferase activity compared with the control mimics when cotransfected with the *IRF2* 3’ UTR reporter plasmid in HD11 cells. In contrast, the inhibition of luciferase activity was attenuated after cotransfection with the mutant-type vector of *IRF2* 3’ UTR and gga-miR-455-5p. Collectively, the luciferase reporter assays demonstrated that gga-miR-455-5p may be the regulator of *IRF2* in chickens.

To investigate the role of gga-miR-455-5p in the regulation of *IRF2*, the mRNA and protein-expression levels of *IRF2* were identified in HD11 cells transfected with the mimic or inhibitor of gga-miR-455-5p. As shown in Figure 8C–H, overexpression of gga-miR-455-5p reduced the cellular expression of *IRF2* at both the mRNA and protein levels, and the miR-455-5p inhibitor enhanced the cellular expression of *IRF2* at both the mRNA and protein levels. These data demonstrated that gga-miR-455-5p directly targeted the 3’ UTR of *IRF2*.

### 2.9. RIP2 Regulated gga-miR-455-5p, IRF2, IFNα, and IFNβ with or without APEC Infection

**Figure 9:**
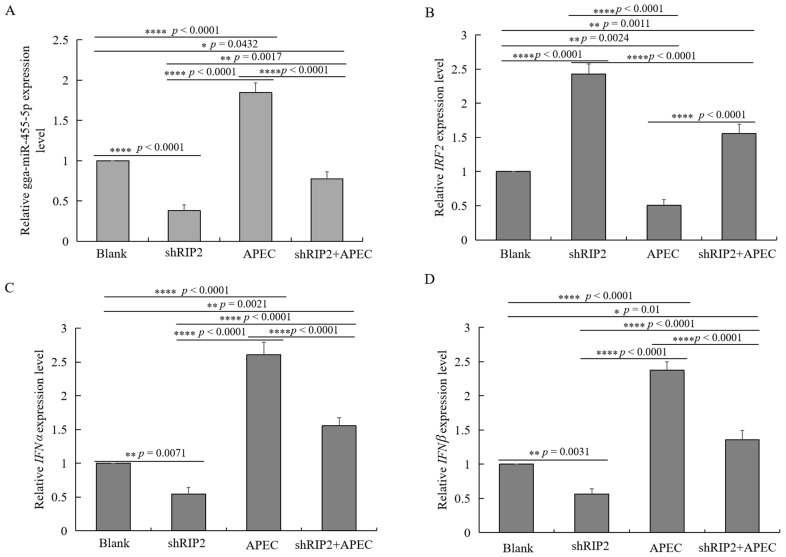
*RIP2* regulates gga-miR-455-5p, *IRF2*, *IFNα*, and *IFNβ* with or without APEC infection. (**A**–**D**) The relative expression level of gga-miR-455-5p (**A**), *IRF2* (**B**), *IFNα* (**C**), and *IFNβ* (**D**) in knockdown of *RIP2* HD11 cells with or without APEC infection. Data are shown as mean ± SD; *n* = 3 independent experiments; * *p* < 0.05, ** *p* < 0.01, **** *p* < 0.001.

To identify the function of *RIP2* in the regulation of gga-miR-455-5p, *IRF2*, *IFNα*, and *IFNβ*, HD11 cells were transfected with *RIP2*-specific small hairpin RNA (shRIP2) with or without APEC infection. The results showed that knockdown of *RIP2* efficiently inhibited the expression of gga-miR-455-5p, *IFNα*, and *IFNβ* with or without APEC infection (Figure 9A,C,D), whereas it significantly increased the expression of *IRF2* (Figure 9B). The results of our study collectively demonstrated that *RIP2* had the ability to regulate gga-miR-455-5p, *IRF2*, *IFNα*, and *IFNβ* with or without APEC infection.

### 2.10. A Model to Explain the Role of RIP2 and the gga-miR-455-5p/IRF2 Axis in APEC Infection

**Figure 10:**
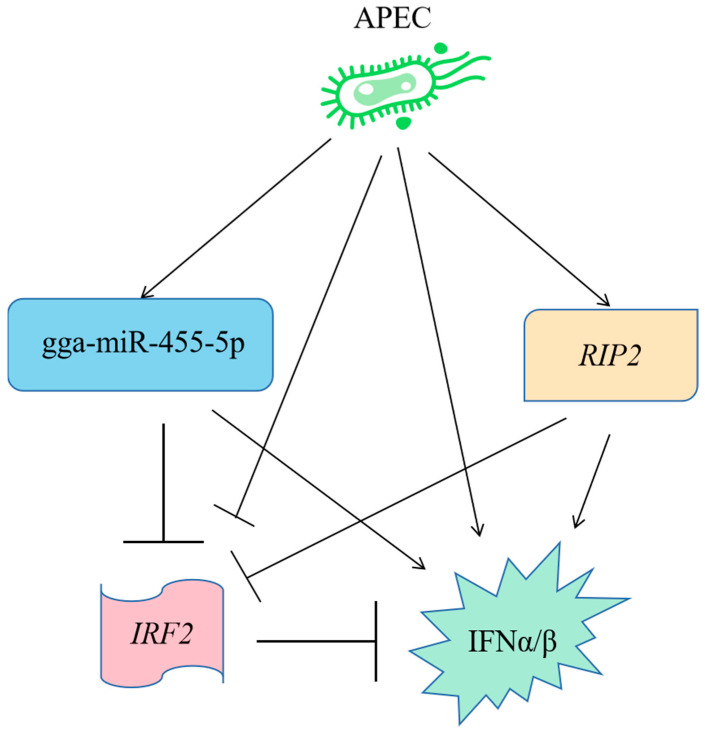
A model to explain the role of ***RIP2*** and the gga-miR-455-5p/IRF2 axis in assisting cellular immune and inflammatory response against APEC infection.

In summary, a novel signaling pathway was identified as involved in the cellular immune and inflammatory response against APEC infection (Figure 10). First, APEC infection could upregulate the expression of gga-miR-455-5p, *RIP2*, *IFNα*, and *IFNβ*, whereas it could decrease the expression of *IRF2*. In addition, overexpression of gga-miR-455-5p decreased cell viability upon APEC infection, while knockdown of gga-miR-455-5p increased cell viability upon APEC infection. Moreover, mechanistic research showed that gga-miR-455-5p downregulated the mRNA and protein levels of IRF2 by directly targeting its 3′ UTR. Furthermore, gga-miR-455-5p enhanced the expression of *IFNα* and *IFNβ* in response to APEC infection. Meanwhile, *RIP2* could positively regulate the gga-miR-455-5p, *IFNα*, and *IFNβ* with or without APEC infection, while it could negatively modulate the expression of *IRF2* with or without APEC infection (Figure 10). Collectively, our results demonstrated that the gga-miR-455-5p related to *RIP2* regulation had the ability to be involved in APEC infection via targeting *IRF2*.

## 3. Discussion

Since miRNAs are involved in regulating gene-expression programs during bacterial infections [26,27], the specific aim of this study was to evaluate the potential role of new miRNAs in the condition of APEC infection, with a particular focus on those that were correlated with *RIP2*. The miRNAs of interest both involved in APEC infection and related to the *RIP2* were identified by analyzing the differentially expressed (DE) miRNAs in both shRIP2 vs. WT and shRIP2+APEC vs. shRIP2.

Of those common identified 70 DE miRNAs in the two comparisons, some of them were related to different kinds of viral or bacterial infections, as well as different cancers. For example, gga-miR-148a-5p was detected in APEC-infection cells (fold change = 1.76) and *RIP2*-silenced cells (fold change = −2.79) in the current study. A recent study reported that gga-miR-148a-5p was significantly downregulated during avian leukosis virus subgroup J (ALV-J) infection and directly targeted *PDPK1* to inhibit the proliferation and cell cycle of ALV-J-infected CEF cells [28]. Similarly, Wu et al. demonstrated that the expression of gga-miR-148a-5p was suppressed in fowl adenovirus aerotype 4-infected hepatocellular cells [29]. Apparently in disagreement, our results showed that gga-miR-148a-5p was upregulated by APEC infection. It seems that gga-miR-148a-5p performs its role in viral and bacterial infections through different molecular mechanisms. Additionally, another miRNA that was correlated with *RIP2* expression appeared worthy of attention; namely, gga-miR-144-5p, which was upregulated in APEC-infection cells (fold change = 2.78) and downregulated in *RIP2*-silenced cells (fold change = −10.27), thus suggesting a possible correlation between it and *RIP2*. To date, there are a few papers in the literature mentioning miR-144-5p; these reported data related to rheumatoid arthritis, cholangiocarcinoma, non-small-cell lung cancer, etc. [30,31,32]. However, they are very few papers that studied this miRNA in chicken. Further studies are needed to clarify the role of gga-miR-144-5p in APEC infection and *RIP2*-knockdown status.

In this study, we also validated the molecular mechanism of gga-miR-455-5p of most interest based on our results, both present and past, and in relation to the data present in the literature. It has been demonstrated that gga-miR-455-5p played an important role in various cancers and virus [33,34,35,36]. For example, miR-455-5p had the ability to promote cell invasion and migration in breast cancer [35]. In chickens, gga-miR-455-5p was downregulated in response to Newcastle disease virus (NDV) infection, and targeted *SOCS3* to inhibit NDV replication [36]. However, the expression pattern of gga-miR-455-5p seems to be different between viral and bacterial infections. In the present study, we found that gga-miR-455-5p was upregulated in APEC-infected cells (fold change = 2.64) and downregulated in *RIP2*-silenced cells (fold change = −3.23). Inhibition of gga-miR-455-5p can markedly reduce the expression of *IL8*, *IL1β*, *IL6*, *IFN*α, and *IFNβ* with APEC infection, indicating gga-miR-455-5p is involved in the regulation of the host immune and inflammatory response against APEC infection.

In addition, the underlying mechanisms by which gga-miR-455-5p modulated the production of proinflammatory cytokines were further investigated. In the current study, we found that gga-miR-455-5p had the ability to enhance the expression of *IFNɑ* and *IFNβ* in response to APEC infection. A similar phenomenon was also detected in NDV infection; that is, gga-miR-455-5p could upregulate the expression of type I interferon [36]. In addition, it was found that the effects of gga-miR-455-5p may be mediated by regulating interferon regulatory factor 2 (*IRF2*). IRF2, a member of the interferon regulatory transcription factor (IRF) family, can compete with IRF1 for DNA-binding sites in IRF-responsive target genes, including interferons alpha (*IFN*α) and beta (*IFNβ*) [37]. Moreover, it has been demonstrated that the type I interferons (IFNα/β) can regulate host immune and inflammatory processes in addition to their antiviral properties in mammals and chickens [38,39,40]. Actually, the type I interferons (IFNα/β) were significantly upregulated after APEC infection, which was consistent with previous APEC studies in bone marrow, blood, and spleen [6,7,8]. In view of the current research results, it seems that gga-miR-455-5p can modulate the inflammatory cytokines by targeting *IRF2* involved in the response to APEC infection. Moreover, we found that knockdown of *RIP2* could not only affect the expression of gga-miR-455-5p, but also the type I interferons (IFNα/β) in chicken HD11 cells. Pandey et al. also proved that the production of type I interferons (IFNα/β) upon *Mycobacterium tuberculosis* infection were significantly decreased in *RIP2*-deficient mice macrophages [41]. Similar results were also found in studied by Lecat et al. and Kim et al. [42,43]. According to the results of this experiment and previous studies, *RIP2* is a required gene to induce the expression of type I interferons (IFNα/β) in response to APEC infection. In summary, *RIP2* can not only directly regulate the production of the type I interferons (IFNα/β), but also indirectly modulate those cytokines via gga-miR-455-5p.

## 4. Materials and Methods

### 4.1. Cell Culture

The chicken macrophage-like cell line HD11 was maintained in RPMI1640 (Gibco, Carlsbad, CA, USA) supplemented with 10% fetal bovine serum (FBS, Gibco, Carlsbad, CA, USA) in a humidified incubator with 5% CO_2_ at 37 °C, and cells were passaged before 80–90% confluence.

### 4.2. Gene Clone, Vector Construction, and Cell Transfection

Small hairpin *RIP2* (shRIP2) plasmid (Table S1), the 3′UTR of *IRF2* and its mutation (Table S2), and gga-miR-455-5p mimics and inhibitor (Table S3) were synthesized by GenePharma (Shanghai, China). The Lipofectamine™ 8000 reagent (Invitrogen, Carlsbad, CA, USA) was used for the cell transfection according to the manufacturer’s instructions. After transfection with shRIP2 for 48 h, cells were treated with or without 0.1 mL 1 × 10^8^ cfu of APEC O78 for 24 h, and collected for total RNA for the miRNA-seq. Cells were transfected with the gga-miR-455-5p mimics or inhibitor for 48 h. Then, cells were collected for RT-qPCR or challenged with 0.1 mL 1 × 10^8^ cfu of APEC O78 for 48 h for further study.

### 4.3. RNA Extraction, Library Preparation, and Deep Sequencing

Total RNA was isolated from cells of the wild-type group (WT), *RIP2*-knockdown group (shRIP2), APEC-challenge group (APEC), and *RIP2*-knockdown with APEC challenge group (shRIP2+APEC) using an miRNA Purification Kit (CWBIO, Beijing, China) according to the manufacturer’s instructions. A total of 3 µL RNA was used as input for miRNA library preparation by using the KC-Digital^TM^ small RNA Library Prep Kit for Illumina^®^ (Catalog No. DR08602, Wuhan Seqhealth Co., Ltd., Wuhan, China) following the manufacturer’s instructions. The kit eliminated duplication bias in the PCR and sequencing steps by using a unique molecular identifier (UMI) of 8 random bases to label the preamplified small RNA molecules. The eluted cDNA library was separated using 6% PAGE gel. Approximately 160 bp bands were isolated, purified, and quantified using Qubit 3.0, and finally sequenced on a Hiseq X-10 sequencer (Illumina) with a PE150 model.

### 4.4. MiRNA-Seq Data Analysis

Raw sequencing data were firstly filtered using fastx_toolkit (version 0.0.13.2, http://hannonlab.cshl.edu/fastx_toolkit/download.html accessed on 18 May 2022) for discarding the low-quality reads, and adaptor sequences were trimmed using cutadapt (version 1.15, https://cutadapt.readthedocs.io/en/v1.15 accessed on 18 May 2022). Clean reads were further treated with in-house scripts to eliminate the duplication bias introduced in the library preparation and sequencing. The consensus sequences from each sample with duplicated removed were mapped to the reference genome of *Gallus gallus 6.0* using bowtie (version 1.1.2, http://bowtie-bio.sourceforge.net/index.shtml accessed on 18 May 2022) with the default parameters. The mirdeep2 package (version 2.0.0.8, https://www.osc.edu/book/export/html/4389 accessed on 18 May 2022) was used to map the reads to the known primary miRNA in the miRBase database and to predict the novel miRNA. The miRNA differentially expressed between groups was identified using the edgeR package (version 3.12.1, http://www.bioconductor.org/packages/release/bioc/html/edgeR.html accessed on 18 May 2022). A cutoff of *p*-value < 0.05 and |fold change| > 1.5 were used to judge the statistical significance of miRNA expression differences. The target mRNA of differentially expressed miRNA was predicted using miRanda (version 3.3a, https://anaconda.org/bioconda/miranda accessed on 18 May 2022). Gene ontology (GO) [44] and Kyoto Encyclopedia of Genes and Genomes (KEGG) enrichment [45] analyses of the targeted mRNA were implemented using KOBAS software (version 2.1.1, http://kobas.cbi.pku.edu.cn/download accessed on 18 May 2022) with a corrected *p*-value cutoff of 0.05 to judge statistically significant enrichment. The miRNA–mRNA coexpression network was constructed using WGCNA (version 1.61, https://cran.r-project.org/src/contrib/Archive/WGCNA accessed on 18 May 2022) with a correlation threshold >0.8 between the miRNA and mRNA.

### 4.5. RT-qPCR Analysis

Total RNA was isolated from cells of different groups using Trizol reagent (Invitrogen, Carlsbad, CA, USA) according to the manufacturer’s instructions. Then, the RNA was reverse-transcribed into cDNA using a reverse-transcription kit (Takara, Dalian, China). The One Step SYBR^®^ PrimeScript^®^ PLUS RTRNA PCR Kit (Takara, Dalian, China) was used for cDNA synthesis. The RT-qPCR was conducted using a SYBR^®^ Premix Ex Taq^TM^ II kit (Takara, Dalian, China) to evaluate the expression levels of *RIP2*, *IL8*, *IL6*, *IL1β*, *IFNɑ*, *IFNβ*, *IRF2*, *GADPH*, U6, gga-miR-455-5p, gga-miR-99a-5p, gga-miR-135a-5p, gga-miR-204, gga-miR-34b-5p, gga-miR-144-5p, gga-miR-18a-3p, gga-miR-26a-5p, gga-miR-147, gga-miR-1306-5p, and gga-miR-148a-5p. Primer sequences are displayed in Tables S4 and S5. The gene-expression levels were normalized to the *GADPH* gene-expression level, and the miRNA expression level was normalized to that of U6. Relative expressions of mRNAs and miRNAs were calculated using the 2^−ΔΔCT^ method as previously described [46].

### 4.6. Western Blotting

Cells from different groups were lysed on ice using 200 μL RIPA buffer (Beyotime Biotechnology, Shanghai, China) for 30 min. Next, the lysis mixtures were centrifuged and the supernatants were collected. A BCA™ Protein Assay Kit (Pierce, Appleton, WI, USA) was used for quantification of proteins. Then, the isolated proteins were subjected to sodium dodecyl sulfate–polyacrylamide gel (SDS-PAGE) and electrophoretically transferred to PVDF membranes. Afterward, membranes were blocked in 5% BSA for 2 h at room temperature and then probed with the primary antibodies at 4 °C overnight. The primary anti-IRF2 (12525-1-AP, Proteintech, Rosemont, IL, USA) was used at a dilution ratio of 1:1000. Then, the membranes were incubated with secondary antibodies tagged with horseradish peroxidase (Sigma-Aldrich, St. Louis, MO, USA) at a 1:10,000 dilution ratio at room temperature for 2 h. Then, immunoblots were visualized using enhanced chemiluminescence (ECL kit, Santa Cruz Biotechnology, Dallas, TX, USA). The blots were visualized using Image Lab™ Software (Bio-Rad, Hercules, CA, USA).

### 4.7. Cell Viability Assay

A Cell Counting Kit 8 (CCK-8) was utilized to determine the viability of cells from different groups (WT, APEC, gga-miR-455-5p mimic + APEC, and gga-miR-455-5p inhibitor + APEC). Cells from each of the four groups were placed in three replicates at a density of 1 × 10^5^ cells per well in a 96-well plate with 100 µL of medium and incubated for 48 h. Then, the cells were incubated for 2 h in 10 µL of CCK-8 solution. Absorbance (optical density (OD)) was assessed at 450 nm using a spectrophotometer.

### 4.8. Luciferase Assay

The luciferase reporter assay was performed as previously described with slight modifications [47]. Briefly, HD11 cells were cotransfected with 500 ng of *IRF2* wild-type/*IRF2* mutation plasmid and 500 ng gga-miR-455-5p of mimics, inhibitor, or miR-NC using Lipofectamine 8000™ transfection reagent (Invitrogen, Carlsbad, CA, USA). The cells were lysed 24 h after transfection, and the luciferase activity in the total cell lysates was measured using a dual-luciferase reporter assay system (Beyotime, Shanghai, China) according to the manufacturer’s instructions.

### 4.9. Statistical Analyses

Statistical analysis was conducted using a one-way ANOVA and Tukey’s honestly significant differences test (HSD; SAS, 2000; Cary, NC, USA) using JMP statistical software (version 15.2.1, SAS Institute, Cary, NC, USA). Data are expressed as the mean ± standard error. The statistical significance was defined at *p* < 0.05. The test results represent the data of four independent experiments.

## 5. Conclusions

In conclusion, this paper presented the first characterization of chicken macrophages’ miRNA expression profile against APEC infection, especially correlated with *RIP2* expression. A total of 93 and 148 DE miRNAs were identified in the comparison of shRIP2+APEC vs. shRIP2 and shRIP2 vs. WT, respectively. Moreover, 262 and 467 target genes were predicted to respectively correspond to those identified DE miRNAs for the two comparisons, and were mainly significantly enriched in tight junctions, focal adhesions, and the ErbB signaling pathway. Many miRNAs, such as gga-miR-148a-5p, gga-miR-144-5p, and gga-miR-455-5p, were detected as involved in APEC infection and related to *RIP2* expression, even though they had not yet been reported to correlate with APEC and *RIP2*. Significantly, gga-miR-455-5p regulated by *RIP2* could directly alter the expression of the target *IRF2* to modulate cytokines and participate in the immune and inflammatory response against APEC infection. In addition, *RIP2* had the ability to directly regulate the expression of the production of type I interferons (IFNα/β). Our findings will deepen the understanding of the mechanisms of the host immune response against APEC infection in chickens through miRNA-induced systems, especially correlated with *RIP2* expression. It is worth mentioning that gga-miR-455-5p might provide a potential target for the treatment of APEC infection and assist in breeding for genetic resistance to APEC in poultry in the future.

## Data Availability

The data are available from the corresponding author upon request.

## Supplementary Materials

The following supporting information can be downloaded at: www.mdpi.com/article/10.3390/ijms23137319/s1.
