# Supplementary material for: Analysis of miRNA Expression Profiling of RIP2 Knockdown in Chicken HD11 Cells When Infected with Avian Pathogenic E. coli (APEC)
Source: Int J Mol Sci. 2022 Jun 30;23(13):7319. doi: 10.3390/ijms23137319 (PMC9266748; doi:10.3390/ijms23137319)
Supplement: Supplementary file 1 [file ijms-23-07319-s001.zip › ijms-1755648-supplementary.pdf]

Legends for the supplementary figures

Figure S1 Characteristics of small RNA in wild type (WT), *RIP2* knockdown (shRIP2), avian pathogenic *E. coli* infection (APEC), and *RIP2* knockdown combined with APEC infection group (shRIP2+APEC). A. Read length distribution statistics in different groups. B. The distribution of reads on the functional elements of *gallus gallus* 6.0 genome in different groups. C. The density distribution of reads on each chromosome for different groups.

Figure S2 miRNA nucleotide bias analysis results. A and C. Proportion of known (A) and novel miRNA (C) first nucleotide bias. B and D. Proportion of known (B) and novel miRNA (D) nucleotide bias at each position.

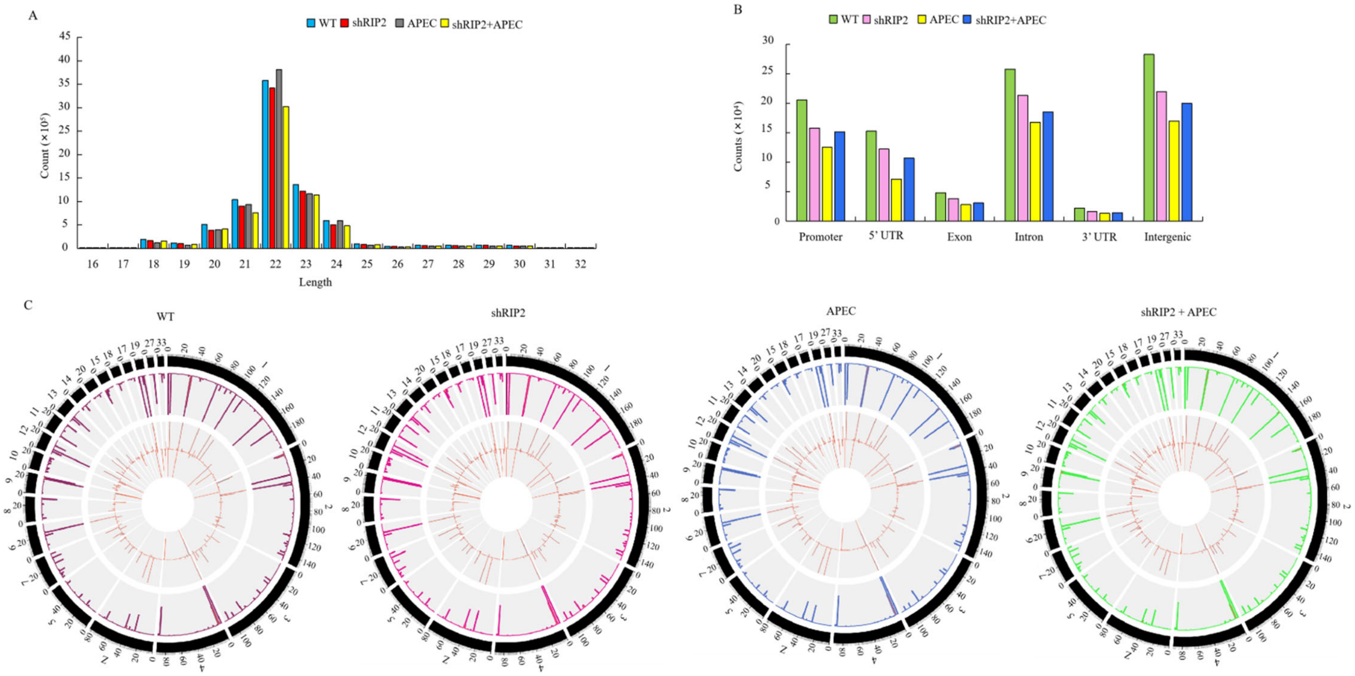

Figure S1 Characteristics of small RNA in wild type (WT), *RIP2* knockdown (shRIP2), avian pathogenic *E. coli* infection (APEC), and *RIP2* knockdown combined with APEC infection group (shRIP2+APEC). A. Read length distribution statistics in different groups. B. The distribution of reads on the functional elements of *gallus gallus* 6.0 genome in different groups. C. The density distribution of reads on each chromosome for different groups.

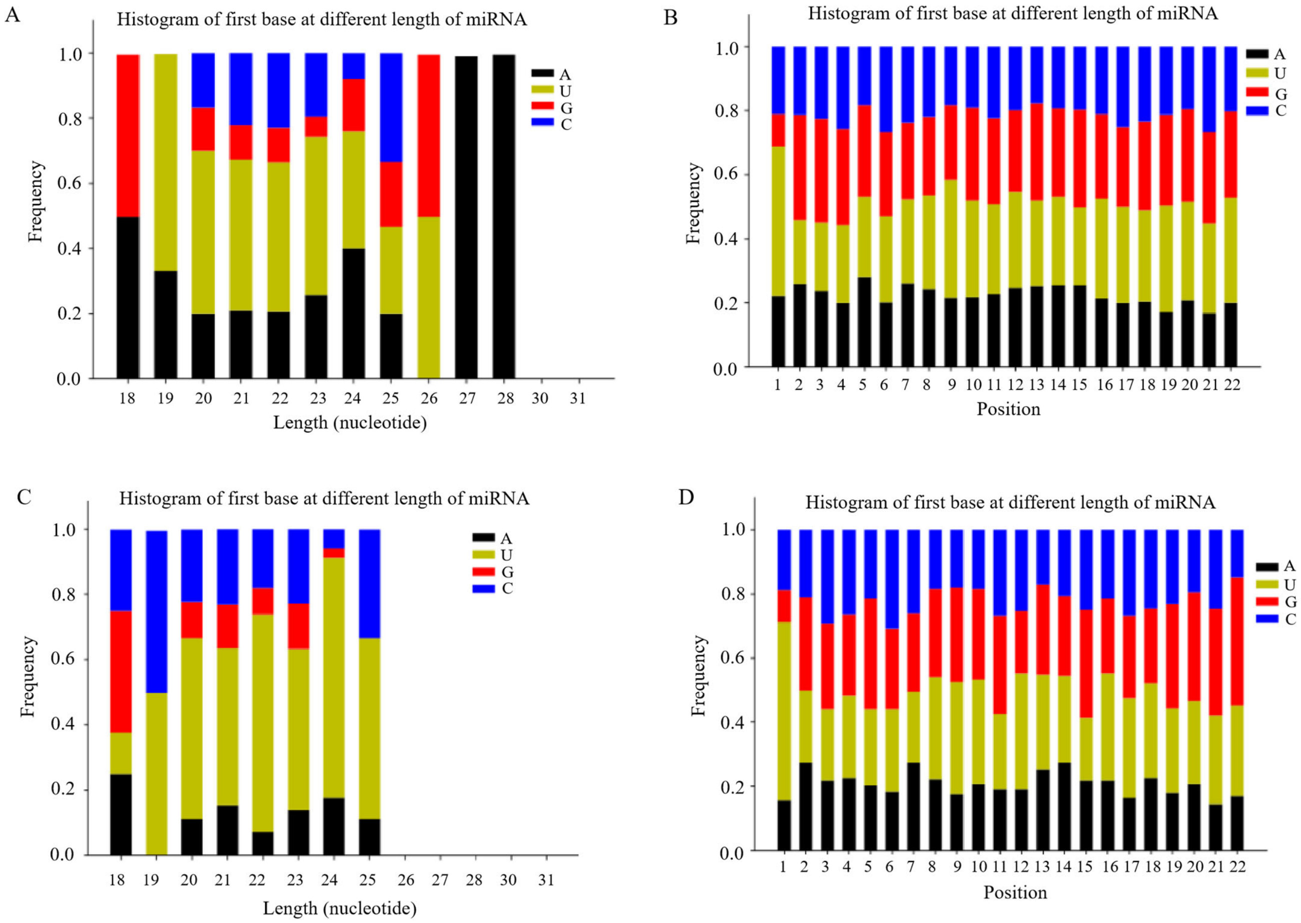

Figure S2 miRNA nucleotide bias analysis results. A and C. Proportion of known (A) and novel miRNA (C) first nucleotide bias. B and D. Proportion of known (B) and novel miRNA (D) nucleotide bias at each position.

Legends for the supplementary tables

Table S1 Characteristics of RNA sequencing data before or after quality control

Table S2 The reads mapping information for each sample

Table S3 shRNA information for *RIP2*

Table S4 The information for wild type and mutant 3′ UTR of *IRF2*

Table S5 The information of gga-miR-455-5p mimics and inhibitor

Table S6 Primers for *RIP2*, *IRF2*, and inflammatory genes in RT-qPCR experiment

Table S7 Primers for candidate miRNAs by RT-qPCR

Table S1 Characteristics of RNA sequencing data before or after quality control

Samples	Raw reads	Clean reads	N%>1%	Adaptor contamine	Low quality	Length < 18 nt or Length > 30 nt
WT_1	24,577,952	11,363,586 (46.23%)	12 (0.0%)	104,886 (0.43%)	387,585 (1.58%)	12,721,883 (51.76%)
WT_2	21,113,215	9,915,189 (46.96%)	10 (0.0%)	75,441 (0.36%)	272,540 (1.29%)	10,850,035 (51.39%)
WT_3	23,425,323	11,007,959 (46.99%)	13 (0.0%)	90,582 (0.39%)	347,961 (1.49%)	11,978,808 (51.14%)
shRIP2_1	20,707,422	10,373,329 (50.09%)	10 (0.0%)	78,042 (0.38%)	286,785 (1.38%)	9,969,256 (48.14%)
shRIP2_2	21,160,012	10,506,782 (49.65%)	7 (0.0%)	82,933 (0.39%)	305,553 (1.44%)	10,264,737 (48.51%)
shRIP2_3	22,966,845	11,490,468 (50.03%)	10 (0.0%)	85,487 (0.37%)	347,839 (1.51%)	11,043,041 (48.08%)
APEC_1	16,497,027	8,985,290 (54.47%)	0 (0.0%)	80,717 (0.49%)	251,258 (1.52%)	7,179,762 (43.52%)
APEC_2	16,843,042	9,152,665 (54.34%)	0 (0.0%)	84,683 (0.5%)	269,021 (1.6%)	7,336,673 (43.56%)
APEC_3	27,907,143	15,601,765 (55.91%)	2068 (0.01%)	129,867 (0.47%)	461,714 (1.65%)	11,711,729 (41.97%)
shRIP2+APEC_1	18,655,185	8,993,311 (48.21%)	0 (0.0%)	84,894 (0.46%)	280,801 (1.51%)	9,296,179 (49.83%)
shRIP2+APEC_2	18,654,422	9,064,973 (48.59%)	0 (0.0%)	76,288 (0.41%)	280,328 (1.5%)	9,232,833 (49.49%)
shRIP2+APEC_3	17,635,198	8,548,952 (48.48%)	0 (0.0%)	78,151 (0.44%)	273,596 (1.55%)	8,734,499 (49.53%)

Note: WT indicates wild type; shRIP2 means knockdown of *RIP2* HD11 cells; APEC represents avian pathogenic *E. coli* infection; shRIP2+APEC indicates knockdown of *RIP2* HD11 cells combined with APEC infection; nt means nucleotide.

Table S2 The reads mapping information for each sample

Samples	Clean unique reads	Clean total reads	Mapped unique reads	Mapped total reads	Mapping rate
WT_1	270,938	8,206,252	237,948	7,972,378	97.15%
WT_2	250,850	7,294,159	220,887	7,083,121	97.11%
WT_3	274,643	8,002,584	237,031	7,736,290	96.67%
shRIP2_1	227,847	6,883,482	201,746	6,615,212	96.10%
shRIP2_2	228,910	6,986,488	202,917	6,714,661	96.11%
shRIP2_3	238,961	7,372,155	211,852	7,084,657	96.10%
APEC_1	170,405	6,199,420	148,566	5,995,491	96.71%

APEC_2	173,199	6,290,667	151,035	6,080,291	96.66%
APEC_3	244,563	9,723,002	209,834	9,392,915	96.61%
shRIP2+APEC_1	220,075	6,526,083	194,078	6,329,463	96.99%
shRIP2+APEC_2	220,372	6,523,876	194,765	6,326,277	96.97%
shRIP2+APEC_3	211,262	6,174,384	186,449	5,988,857	97.00%

Note: WT indicates wild type; shRIP2 means knockdown of *RIP2* HD11 cells; APEC represents avian pathogenic *E. coli* infection; shRIP2+APEC indicates knockdown of *RIP2* HD11 cells combined with APEC infection.

Table S3 shRNA information for *RIP2*

ID	5’	stem	loop	stem	3’
NC-F	GATCC	GACGAAAGTCAAGACTTCTATTCAA	TTCAAGAGA	TTGAATAGAAGTCTTGACTTTCGTC	TTTTTTG
NC-R	AATTCAAAAAA	GACGAAAGTCAAGACTTCTATTCAA	TCTCTTGAA	TTGAATAGAAGTCTTGACTTTCGTC	G
shRNA1-F	GATCC	ACAGTGATAGAAACCACCTTCTAAA	TTCAAGAGA	TTTAGAAGGTGGTTTCTATCACTGT	TTTTTTG
shRNA1-R	AATTCAAAAAA	ACAGTGATAGAAACCACCTTCTAAA	TCTCTTGAA	TTTAGAAGGTGGTTTCTATCACTGT	G
shRNA2-F	GATCC	GACTTAAGTGAAACCAGCTTATCAA	TTCAAGAGA	TTGATAAGCTGGTTTCACTTAAGTC	TTTTTTG
shRNA2-R	AATTCAAAAAA	GACTTAAGTGAAACCAGCTTATCAA	TCTCTTGAA	TTGATAAGCTGGTTTCACTTAAGTC	G
shRNA3-F	GATCC	ACAAACAGCTAGGACTTCAACCTTA	TTCAAGAGA	TAAGGTTGAAGTCCTAGCTGTTTGT	TTTTTTG
shRNA3-R	AATTCAAAAAA	ACAAACAGCTAGGACTTCAACCTTA	TCTCTTGAA	TAAGGTTGAAGTCCTAGCTGTTTGT	G

Table S4 The information for wild type and mutant 3′ UTR of *IRF2*

Name	Synthesized Sequence
IRF2 wild type (3′ UTR)	GCTAGCaatattgtacatagtgcagtactagcaacgttgcagtctgcttctgcaccttattaagaaagcacttacaaaaggcctttt attaccttgctctacttaatggcacattgaaggtccctccccacctgtattcttttccaaggctattcttgctaaagtgtctttaaagaatag tggaaaggaaagaaagttaaaacttcCTCGAG
IRF2 mutation (3′ UTR)	GCTAGCaatattgtacatagtgcagtactagcaacgttgcagtctgcttctgcaccttattaagaaagcacttacaaaaggcctttta ttaccttgctctacttaatttacacgtgaaggtccctccccacctgtattcttttccaaggctattcttgctaaagtgtctttaaagaatagt ggaaaggaaagaaagttaaaacttcCTCGAG

Table S5 The information of gga-miR-455-5p mimics and inhibitor

Name	Sequence
gga-miR-455-5p mimics	UAUGUGCCCUUGGACUACAUCG
gga-miR-455-5p mimic negative control	UCACAACCUCCUAGAAAGAGUAGA
gga-miR-455-5p inhibitor	CGAUGUAGUCCAAGGGCACAUA
gga-miR-455-5p inhibitor negative control	UCUACUCUUUCUAGGAGGUUGUGA

Table S6 Primers for *RIP2*, *IRF2* and inflammatory genes in RT-qPCR experiment

Name	Gene ID	Forward primer (5′-3′)	Reverse primers (5′-3′)
RIP2	420215	CTCGAACCAGTCCTGAGAACG	AAGCGGATGTTTCCTCTTGG
IRF2	396115	TGTTTGCCAGGTAGTAGAGG	ACGCTGTCGGTCGTTTCAC
IL8	395872	GAGTTCACTGACCACCCT	TGCCTGAGCCATACCTTT
IFNα	396398	GACAGCCAACGCCAAAGC	GTCGCTGCTGTCCAAGCATT
IFNβ	554219	CGTCACCAGGATGCCAACT	AGCCGTATTCTGAAGGAGC
IL6	395337	GGAGAAATGCCTGACGAA	GGATTGTGCCCGAACTAA
IL1β	395196	CGCTTCATCTTCTACCGC	CCAGTCACAATAAATACCTCCA

Table S7 Primers for candidate miRNAs by RT-qPCR

Name	Primer sequence(5′-3′)
gga-miR-455-5p	TATGTGCCCTTGGACTACATCG
gga-miR-99a-5p	AACCCGTAGATCCGATCTTGTG
gga-miR-135a-5p	TATGGCTTTTTATTCCTATGTGA
gga-miR-204	TTCCCTTTGTCATCCTATGCCT
gga-miR-34b-5p	CAGGCAGTGTAGTTAGCTGATTG
gga-miR-144-5p	GGATATCATCATATACTGTAAG
gga-miR-18a-3p	ACTGCCCTAAGTGCTCCTTCTG
gga-miR-26a-5p	TTCAAGTAATCCAGGATAGGC
gga-miR-147	GTGTGCGGAAATGCTTCTGC
gga-miR-1306-5p	ACCACCTCCCCTGCAAACGTCCAGT
gga-miR-148a-5p	AAAGTTCTGTGACACTCAGACT
